# A qualitative exploration of malaria operational research situation in Nigeria

**DOI:** 10.1371/journal.pone.0188128

**Published:** 2017-11-15

**Authors:** IkeOluwapo O. Ajayi, Maduka D. Ughasoro, Akintayo Ogunwale, Oluwaseun Odeyinka, Obafemi Babalola, Salami Sharafadeen, Al-Mukhtar Y. Adamu, Olufemi Ajumobi, Taiwo Orimogunje, Patrick Nguku

**Affiliations:** 1 Nigeria Field Epidemiology and Laboratory Training Programme, Abuja, Nigeria; 2 Department of Epidemiology and Medical Statistics, Faculty of Public Health, College of Medicine, University of Ibadan, Ibadan, Nigeria; 3 Department of Paediatrics, University of Nigeria Enugu Campus, Enugu, Nigeria; 4 Department of Health Promotion and Education, Faculty of Public Health, College of Medicine, University of Ibadan, Ibadan, Nigeria; 5 Department of General Studies, Oyo State College of Agriculture and Technology Igboora, Igboora, Oyo State; 6 Department of Medical Microbiology and Parasitology, Faculty of Clinical Sciences, Bayero University, Kano, Nigeria; 7 African Field Epidemiology Network, Abuja, Nigeria; 8 National Malaria Elimination Programme, Abuja, Nigeria; Centers for Disease Control and Prevention, UNITED STATES

## Abstract

**Background:**

Malaria, remains one of the leading causes of high morbidity and mortality in Nigeria despite implementation of several public health interventions for its control. Operational limitations and methodological gaps have been associated with malaria control interventions and research, and these have necessitated the need for a well-tailored Malaria Operational Research (MOR) agenda. However, there is paucity of evidence-based information on relevant stakeholders’ experience, awareness, perceptions and use of MOR and suggestions on setting MOR agenda. As part of a larger study to provide data for national MOR agenda setting, we assessed the MOR research situation from the perspectives of key stakeholders in Nigeria and contribution of MOR to the malaria elimination agenda

**Methods:**

We conducted key informant interviews among 40 purposively selected stakeholders from the six geo-political zones in Nigeria. Data was collected using a pre-tested key informant interview guide which comprised issues related to experience, awareness, use of MOR and MOR needs, and suggestions for MOR. We conducted a detailed content analysis.

**Results:**

Half of the participants had participated in MOR. Participants perceived MOR as important. Only few were aware of existing framework for MOR in Nigeria while above half expressed that MOR is yet to be used to inform policy in Nigeria. Participants identified several MOR needs such as development of improved diagnostic techniques, and interventions for promoting early diagnosis, prompt treatment and quality programmatic data. Participants opined the need for country-specific prioritised MOR agenda that cut across malaria thematic areas including malaria prevention and case management. Participants suggested the involvement of various stakeholders and multi-disciplinary approach in setting MOR.

**Conclusion:**

Although some stakeholders have been involved in MOR, it is still rarely used to inform policy and several needs exist across thematic areas. A broad-based stakeholder involvement, multi-disciplinary approach to agenda setting and its wide dissemination have been suggested.

## Introduction

Malaria remains a major public health problem in Nigeria and other sub-Saharan African countries. Under-five mortality is estimated at 128 per 1,000 live births and maternal mortality is estimated at 576 per 100,000 live births [1]. Malaria is transmitted throughout Nigeria, with 97% of the population at risk [2]. The unacceptable impact of the disease on the health, development and economic indices of endemic countries stimulated global interest in scaling up malaria control with the aim of progressing towards elimination in each country and ultimately eradication [3, 4]. In an effort to reduce the malaria scourge, several public health strategies and tools have been implemented through various programmes in Nigeria. There are several interventions that have been tested in various malaria endemic countries and found effective as well as recommended to improve malaria control. While some of these interventions have been evaluated for effectiveness in the Nigerian context using operational research [5], there is still the need to identify many more interventions that could be relevant and scaled up to achieve the National Malaria Strategic Plan of eliminating malaria in Nigeria.

Current malaria control and elimination programmes face remarkable heterogeneity of transmission dynamics of malaria in endemic areas, including differences in parasite, vector, human, social, and environmental factors. Operational limitations include underperforming health services, lack of political will, insufficient financial, social and human resources, and for some areas, inadequate tools to interrupt transmission given an exceedingly high force of transmission [6]. Each country presents different combinations of these problems and their determinants. In order to increase the chance of success of any malaria intervention, the planning and implementation must be based upon proper epidemiological analysis and application of interventions that address specific needs of localities and countries [7]. There is thus the need to complement the current research agenda primarily directed towards reducing morbidity and mortality, with one; that aims to identify key knowledge gaps and will define the strategies and tools that will result in further reduction in the burden of malaria.

Operational research is one of the approaches for identifying gaps and areas of malaria control that need to be enhanced as well as for evaluating effectiveness of intervention for possible scale up. Operational research as defined by Higgins et al. 2011 is 'the search for knowledge on interventions, strategies, or tools that can enhance the quality, effectiveness, or coverage of programmes in which the research is being done' [8]. Employing malaria operational research (MOR) findings in the planning of national malaria control programmes is gaining increased attention [4]. Interventions on vector control, case management and surveillance in a control programme may require modification to be applicable in an elimination programme. Operational research can be very instrumental in providing answers to “what changes”, “where” “why” “when” and “how” these changes should be implemented to ensure smooth transition from control to elimination. However, malaria operational research has continued to receive little attention and the relevance of the ones being conducted to ensure the needed progress from malaria control to elimination is not certain. The National Malaria Elimination programme (NMEP) in Nigeria as part of her strategic plan to set MOR agenda, developed MOR priority topics in the past [2]. However, poor attention has been paid to exploring the priority research topics by researchers and malaria control implementers in Nigeria. In addition, there is paucity of information on perception and experience of researchers in conducting operational research. Thus, as part of a larger study to provide data for National Malaria Operation Research Agenda Setting, we assessed the MOR research situation from the perspectives of key stakeholders in Nigeria using qualitative method. The findings of this study will be useful in planning a sustainable malaria operational research agenda towards eliminating malaria in the nation.

## Methods

### Study setting

Nigeria is the most populous country in Africa with an estimated annual growth rate of about 3.2% and a projected total population of approximately 195 million for 2017. It comprises six geopolitical zones, 36 states (plus the Federal Capital Territory of Abuja), and 774 LGAs. Nigeria like other topical countries has climatic conditions which favour the survival of mosquito vector species and malaria transmission. The duration of the transmission season decreases from year-round transmission in the south to three months or less in the north. *Plasmodium falciparum* is the predominant malaria species. The Federal Ministry of Health has a division for malaria control, the NMEP, which coordinates all activities on malaria control in the country. The NMEP collaborates with development partners and other related organisations to plan malaria control activities. This study was conducted by Nigerian Field Epidemiology and Laboratory Training Programme (NFELTP) in collaboration with NMEP.

### Study design

An exploratory study was conducted among stakeholders and researchers who are working on malaria research and related control programmes in Nigeria using Key Informant Interviews (KII) from October to November 2016. The respondents were development partners, policy makers, programme managers and malaria researchers especially those based in the universities and research institutes.

### Selection of key informant interviewees

The KIIs were conducted among forty purposively selected participants based on their experience in malaria programmes and research, institutional affiliations and thematic areas of interest or focus. Participants were drawn from all the six geo-political zones in Nigeria. The participants were contacted through e-mail or telephone and appointments for interviews were sought. The list of development partners collaborating with NMEP, their malaria programme managers, and that of state malaria programme managers were obtained from the NMEP. Malaria researchers were selected from a database of relevant organisations including NMEP and some by snow-balling approach.

#### The process of key informant interviews

The interviews were conducted using a pretested KII guide developed for the study. The guide sought information on issues including concept of MOR, perception of MOR, capacity for MOR and needs relating to MOR. The guide also covered the participants’ experience on collaborating on MOR, and suggestions they have for the process of setting a MOR agenda.

Six members of the research team comprising consultants/medical doctors and field epidemiologists who had previous experience in qualitative research conducted the key informant interviews in pairs (3 teams). These individuals underwent a three-day training prior to the conduct of the study. The training comprised acquaintance with the data collection instrument. There were also practical sessions on interviewing skills—skills of probing, recording responses, and note-taking, as well as transcription of recordings. The interviews were conducted by a pair of the research experts each in the place and time of choice of interviewees. All the interviews were audio-tape recorded and notes were taken. The average time spent on an interview was 45 minutes. The interviews were held to the point of saturation.

### Analysis of qualitative data

Interview recordings were transcribed daily after the field work. The transcriptions were revalidated by a set of eight trained residents and graduates of the NFELTP. Thereafter the transcribed notes were entered into the computer using ATLAS Ti (version 7.5). Codes were developed and completed by a single coder. Both deductive and inductive approaches were used for coding. The study objectives served as the basis of coding and labels of codes (nodes) were in “tree nodes.” Similar codes were merged and duplicates were removed. The codes and corresponding quotes that were generated were checked thoroughly and presented to a team of experienced researchers for review and contributions.

Themes were generated based on the following: (a) the KII guide (b) sample quotes from transcripts (c) comments, reflections, and underlying meanings based on non-verbal transcripts; and (d) group reflections (contributions from members of the research team). The transcript of each interviewee was thoroughly read and examined theme by theme in comparison with that of other interviewees to identify relevant texts, repeating words, and phrases. For each theme, common and peculiar trends as well as similar and divergent opinions were noted. The various trends, opinions and perspectives of the interviewees together with their identified related verbatim quotes were used to produce a narrative and summary of the findings.

### Ethical considerations

Ethical approval for the study was obtained from the National Health Ethical Review Committee and the joint Ethics Review Committee of the University of Ibadan and University College Hospital, Ibadan. Written informed consent was obtained from the participants. Confidentiality of the information provided by the participants was ensured.

## Results

### Socio-demographic profile of participants

The median age of participants was 45 years with a range of 33 to 64 years. Of the 40 participants, 33 (82.5%) were males. The participants were of multi-disciplinary background. The highest proportions (35.0%) of participants were from universities and research institutions. Participants were of various designations, including directors (15.0%), consultants (15.0%) and senior programme managers (15.0%). Some (7.5%) participants had dual role as professor of university and consultant in teaching hospitals (7.5%) and a few (2.5%) were only professors in universities. The area of malaria related research and programmes the participants specialised in include programme management (30.0%), case management (30.0%) and vector control (20.0%). Details of the socio-demographic information on participants are shown on Table 1.

**Table 1:** Socio-demographic profile, affiliation, designation and research focus of participants [N = 40].

Characteristics	Frequency	Percentage
Age group* **(years)**		
≤40	6	17.1
40–49	18	51.4
50–59	8	22.9
≥ 60	3	8.6
**Sex**		
Male	33	82.5
Female	7	17.5
**Institutional Affiliation**		
Universities/Research Institutes	14	35.0
Non-government organisations	10	25.0
Academia/Teaching Hospital	6	15.0
Federal/State Ministries of Health	6	15.0
Multi-national and International Organisations	3	7.5
Local Government	1	2.5
**Position/Designation**		
Director	6	15.0
Consultant	6	15.0
Senior Programme Manager	6	15.0
Senior Lecturer/Senior Research	5	12.5
Fellow	4	10.0
Professors and Consultants	3	7.5
Country Director/Head	3	7.5
Senior Technical Specialist	3	7.5
Professor	2	5.0
Executive Secretary	1	2.5
Roll-back Malaria Focal Person	1	2.5
**Focus in Malaria Research/Programme**		
Programme Management	12	30.0
Case Management	12	30.0
Vector Control	8	20.0
Malaria Prevention Research	4	10.0
Malaria in pregnancy	2	5.0
Advocacy, Communication and Social Mobilisation	1	2.5
Chemo-prevention	1	2.5

*Missing data were removed from the analysis

### Perception of malaria operational research

The participants viewed malaria operational research as a type of scientific research that focuses on identifying and resolving problems and gaps relating to malaria programmes and interventions with a view to achieving improved outcomes.

“*Operational research is a type of research that is conducted to improve outcomes*, *to understand how services and programmes are being implemented and also to identify where there are gaps and areas to improve upon*.”

*(Participant 38*, *Abia State*, *Public Health Physician)*

All the participants considered malaria operational research important and stated that it will contribute immensely to malaria elimination in Nigeria.

“*Malaria operational research agenda will help to know where to go or where to start from*, *because there are so many issues in malaria*, *so many intervention methods*, *physical control methods and other controls*, *like this chemoprophylaxis*, *intermittent preventive treatment of malaria in pregnancy and others*.”

*(Participant 6*, *Kano State*: *Ministry of Health)*

### Awareness on existing framework that guide malaria operational research in Nigeria

Most of the participants were not aware of any existing framework that guides MOR in Nigeria. About a third mentioned affirmatively that there was no existing framework that guides MOR in Nigeria and ten others expressed that they could not comment.

“*To the best of my knowledge*, *I am not aware of any*, *even though I have gone through the malaria strategic plan*.”

*(Participant 38*: *Abia State*: *Public Health Physician)*

### Availability of capacities to identify country-specific needs for MOR

All the key informants, except three persons, opined that Nigeria has the capacity to identify country-specific needs for MOR. Participants stated that the country has abundant human resources and training institutions that could help in achieving this. Some participants, however, raised concerns about the inadequacies of infrastructural and financial resources for this purpose.

“*There are trained personnel who cut across bodies including public service research institutions*, *and tertiary institutions to identify country-specific needs for MOR*.”

*(Participant 4*: *FCT*: *Ministry*, *Disease Control Programme)*

“*We may have deficiency in funding*, *but I believe that the personnel*, *human resources and the competence to carry out MOR are available*. *And tools are also available*.”

*(Participant 38*: *Abia State*: *Public Health Physician)*

### Experience and collaboration on MOR

Half of the key informant interviewees affirmed that they have been involved in MOR collaborations and interventions and almost half of these were researchers from universities. Many health professionals working in non-governmental organisations (NGOs) also collaborated in various MOR. The participants were involved in various areas of MOR collaborative researches and interventions which included malaria vector surveillance and insecticide resistance monitoring, case management of malaria among under-five and evaluation of the effectiveness of the long-lasting insecticidal net (LLIN). Most of the MOR collaborative researches and interventions were large-scale multi-center studies and were funded by international bodies and donors.

### Malaria operational research needs in Nigeria

The specific needs relating to malaria diagnosis included need for development of improved diagnostics techniques, promotion of adoption of use of rapid diagnostic tests (RDTs) among healthcare workers in private healthcare practice and patent medicine vendors, design of studies that can improve the diagnostic accuracy of RDTs, development of interventions and strategies for promoting early diagnosis, prompt treatment of malaria cases and LLIN use. The need to promote and expand home-based case management was also identified.

“*There is need to carry out research or operational research in area of diagnostic accuracy of RDT*, *although some have been done in the past comparing microscopy with RDT*. *This should address the RDT positive cases that are negative on microscopy and vice visa*.”

*(Participant 24*: *FCT*: *Programme manager)*

“*Increasing access to early diagnosis will enhance prompt treatment*. *This is actually what needs to be done and improved upon using malaria operational research approach*.”

*(Participant 38*: *Abia State*: *Public Health Physician)*

“*Home-based case management needs to be expanded and the people in in the sub-urban areas should key into it*.”

*(Participant 32*: *Anambra State*: *University Researcher)*

“*There is need for operational research that can help us to provide information to guide revision of the distribution strategy to promote the utilization of LLIN*.”

*(Participant 31*: *International Organisation*: *Programme Director)*

“*There is need to attend to the issues of LLINs being uncomfortable to use especially due to heat and other wrong perceptions*.”

*(Participant 19*: *FCT*: *NGO*: *Country Director)*

Design of effective social behavioural change communication (SBCC) interventions that can promote the use of ACTs amidst socio-cultural barriers and existence of fake ACTs and MOR for effective vector control were identified.

“*However*, *we also need to find out why some providers are reluctant to use ACT*, *because we see people still give monotherapy*, *instead of using ACT; I think we also need to*, *probably come up with SBCC strategies that can address that*.”

*(Participant 18*: *FCT*: *NGO*: *Programme Manager)*

“*We need more research studies to know the distribution of both fake and genuine drugs and then the efficacy of the drug too that is the area I think we should concentrate on*.”

*(Participant 24*: *Abuja*: *Programme manager)*

“*In terms of vector control*, *some of the strategies that we use are conventional but in most of the advance country improved technology is being used*. *There is a need to introduce advanced technology such as biological control as mosquitoes are developing resistance*. *In addition*, *teaching of vector control should be included in school curriculum*.”

*(Participant 1*: *Osun*: *University Researcher)*

“…*we need a very serious and well-designed operational research to determine the source(s) of vector resistance in Nigeria*.”

*(Participant 24*: *FCT*: *Programme manager)*

The needs identified for MOR with regards to programme management include improving the quality of our malaria data and its use for programme planning and implementation, recruitment of qualified and competent staff for programme management and use of qualitative research methodology for conducting malaria research.

“*We need to get the right people with the right management and right skills*. *We need to put the round peg in the round hole*.”

*(Participant 4*: *FCT*: *Ministry*: *Disease Control Programme)*

“*The need for good data is becoming very glaring and people are relying more on data in their implementation of activities*…. *there is thus the need to improve the quality of our malaria data and its use*.”

*(Participant 18*: *FCT*: *NGO*: *Programme Manager)*

One of the key informants emphasised the need for multiple approaches for ensuring effective malaria control programmes.

“*We need multiple approaches to diagnosis*, *treatment*, *and prevention with everything integrated; so that as parasites mutate*, *as mosquitoes change habit*, *as people’s drug use habit change*, *one of the approaches will still be effective to deal with the issue of malaria control*.”

*(Participant 5*: *Oyo State*: *University Researcher)*

### Perceptions relating to the contents of national MOR agenda

The key informants acknowledged that most of MOR studies implemented by the NMEP may not be addressing Nigerian indigenous MOR needs and there is a need to develop country-specific prioritised MOR agenda. Their responses revealed further that “*there is need to set and prioritise content of Nigerian National MOR agenda (NMORA) to reflect the areas of needs and should be based on prior needs assessment*.” ***(****Participant 13*: *FCT*: *NGO*: *Programme Manager****)***

The interviewees opined that the content of a national MOR research agenda should reflects the priority areas of needs and should be based on needs assessment for MOR in Nigeria. In addition, the contents should be tied to the objectives, strategies and interventions that are of the focus of NMEP. It was also suggested that the resources and factors needed to support successful implementation of MOR as well as potential barriers or hindrances that could affect the achievements of identified targets for MOR be included in the agenda.

“*The content of the agenda should be pragmatic*, *it should be country need-driven and a research database should be set up*.”

*(Participant 15*: *Rivers State*: *University Researcher)*

Two key informant interviewees mentioned that the content of a national MOR agenda should capture clearly defined research questions and the associated challenges and strategies that could be used to address the challenges, as well as timeline and targets among other issues.

“*I would like answers to these questions*, *‘what do we want to achieve*, *what are the implementation strategies*, *what are the potential problems that could hinder implementation and what will ensure that we achieve our targets*?”

*(Participant 39*: *FCT*: *NGO*: *Director*, *monitoring and evaluation (M&E))*

“*What I expect to see is a good guided policy with target timeline that would help strengthen human capacity*, *infrastructure and then guide implementation in a directed manner*.”

*(Participant 22*: *Rivers State*: *University Researcher)*

“*There should be a mechanism to monitor how effective are the different operational research studies in addressing malaria related needs and gaps*.”

*(Participant 31*: *International Organisation*: *Programme Director)*

“*The agenda should include a standard operating procedure for all stakeholders so the approach to the projects will be similar; you know if you use the similar approach for all these operational research studies*, *we can now compare our findings*.”

*(Participant 23*: *Oyo State*: *University Researcher)*

“*I will like to see the issue of getting and securing political will and commitment from government been captured well in the agenda*.”

*(Participant 4*: *FCT*: *Ministry*: *Head*, *Disease Control Programme)*

The key informant interviewees noted that based on the current malaria situation in Nigeria, the focus of the MOR agenda should cut across all malaria thematic areas but there should be considerable emphasis on malaria prevention and case management.

### Sustainability and capacity building in MOR in Nigeria

The respondents highlighted that involvement of all categories of stakeholders and experts and use of multi-disciplinary approach will be relevant in the process of setting agenda and conducting MOR.

“*The NMEP*, *key partners including donors*, *government ministries and other implementing partners including those working at the community level as well as other stakeholders should be involved in the process of setting the MOR agenda*.”

*(Participant 28*: *FCT*: *International Organisation*: *M & E Director)*

It was suggested that the MOR agenda should be widely disseminated to all MOR stakeholders, be revised regularly and the MOR should be adequately funded and implemented.

The study revealed the need to focus on capacity building for MOR in Nigeria. It was identified that creating increased awareness about malaria operational research, encouraging collaborations with other researchers, institutionalising initiatives on malaria operation research and mentoring of young or upcoming researches could help in achieving capacity building for MOR in Nigeria.

“*Organising training programmes for young ones particularly on malaria operation research can help to address the problem of malaria control in Nigeria*. *Training and capacity development initiatives should be organised for people from different sections of the country*…”

*(Participant 23*: *Oyo State*: *Researcher)*

### Use of MOR to inform policy change

Participants had divergent opinions about the use of MOR to inform policy change. Twenty-five participants opined that MOR and other forms of malaria research are often not used to inform policy change. A frequent response is that disconnect or gaps often exist between policy makers and researchers which often make it difficult to use the outcome of MOR to inform policy in Nigeria.

“*In the past few years the absence of link between researchers and policy makers has been serious concern*. *One of the cardinal objectives of the United State President’s Malaria Initiative is to generate evidence-based information that can inform policy*.”

*(Participant 24*: *FCT*: *Programme manager)*

Five participants asserted that the use of MOR to inform policy change is minimal and policy makers are usually not interested in the use of research outcomes to inform polices on malaria.

“*The use of MOR to inform policy is low because most of the things researchers push forward from research reports should have reflected in policies*. *If the reports are used*, *the country won’t have much challenges with malaria control strategies*.”

*(Participant 25*: *Lagos State*: *University Researcher)*

On the contrary, a quarter of the participants affirmed that use of MOR to inform policy change is gaining popularity as many MOR outcomes are now being used to take policy decisions especially at national level.

“*Research outcomes are being used to inform policy most especially at the national level*. *At the national level it helps to give policy direction*.”

*(Participant 2*: *Oyo State*: *Ministry of Health)*

“*I think stakeholders are beginning to use MOR to inform policy*. *For example*, *I can tell you that the result of a research we carried out on the feasibility of the diagnosis using RDTs which was an operational research was presented at the National Council on Health*, *and yielded a policy change concerning the Patient Medicine Vendors…*. *So increasingly we have this phenomenon happening but it’s not happening enough*, *policy makers need to link with the people who turn evidence-based data out of these research studies*.”

*(Participant 13*: *FCT*: *NGO*: *Programme Manager)*

## Discussion

In this study, participants perceived MOR to be very important in giving directions to aspect of malaria control to focus on, in order to achieve malaria elimination. This perception could be a reflection of the participants’ experience in malaria control programmes and research, as half had been involved in MOR.

The results of this study showed that university-based researchers topped the lists of those involved in malaria MOR, followed by stakeholders in NGO. This finding is similar to that found in the recent five-year review of MOR projects which showed an extensive and active involvement of research institutes as well as funding partners in MOR, and a limited involvement of National Malaria Control Programmes [5]. However, we found low participation of development or funding partners in our study. The active participation of NGOs in MOR in Nigeria is commendable and could actually be a reflection of support from development and funding partners who do not directly engage in research. The involvement of NMEP in MOR is critical to achieving the elimination goal as the programme stands to target effort of MOR at local needs as the programme officers have comprehensive knowledge of the malaria situation in the country and are held responsible for the implementation of control and elimination activities [5]. This involvement will facilitate the uptake of research findings into policies and programme activities of NMEP; though this has been found to be on the increase, it remains deficient. Developing partners are involved in research, thus could support NMEP with wide dissemination of MOR priorities and facilitate conduct of studies on the priorities as well as the subsequent translation of the study findings into policy and practice.

The planning and implementation of a malaria control programme must be based on epidemiological analysis and application of interventions suitable to specific localities or countries [9, 10]. In this study, we explored the current focus of operational research among researchers and areas of need for MOR in Nigeria. The areas of focus of the interviewees’ research projects varied. The most mentioned areas included malaria vector control, case management and use of RDTs. Some of these research studies were funded by development partners, including President’s Malaria Initiative, United States Agency for International Development and World Health Organisation (WHO), and a few were carried out in collaboration with other countries and NMEP. Some of these areas were the focus of studies reviewed by Zhou *et al*. 2014 [5]. However, there remain many yet unanswered research questions that are critical to move forward with malaria elimination [10]. The need to identify MOR gaps and needs, as well as prioritise the needs into a set agenda was alluded to by interviewees in this study and other related study [11].

In this study, the specific needs for the different areas of malaria control were enumerated. These include promotion of use of diagnostic techniques and ACTs, improve case management including scaling up of community management and development of improved diagnostics with respect to case management. Interestingly, the need for integrated approach and building capacity especially in the quality of malaria data and its use was also emphasised. The identified areas are in line with main pillars of the Global Technical Strategy of Malaria 2016–2030 which has the supporting element of harnessing innovation and expanding research to ensure that existing interventions are applied effectively and efficiently in different contexts, and innovation is deployed appropriately and to maximum effect [11, 12].

Findings of this study and reviewed literature suggest that operational research, though recognised as critically important to the success of strategies for malaria control and elimination and identifying areas of needs; it is not commonly undertaken or published. The Global Fund’s Technical Review in 2007, noted that operational research was often missing in most proposals [13]. In this study, one of the interviewees mentioned that operational research is usually mentioned in the country’s malaria strategic plan but was not aware of an existing framework. The NMEP has between year 2011–2014 held stakeholders workshops to develop MOR priority research questions and listed its priorities in OR to improve malaria control [14]. However, these documents were not put in the public domain.

As to what should be in a national MOR agenda, our interviewees mentioned that it should be based on needs assessment for MOR, should have a section that identifies the country’s priority areas of need, clearly capture defined research questions, and be tied to objectives, strategies and intervention focus of National Malaria Strategic Plan among other suggestions. These suggestions are in line with the inputs of OR agenda of other programmes [15]. One other aspect of MOR agenda explored is the sustainability and capacity building for MOR. The interviewees emphasised the use of multi-disciplinary approach to setting the agenda and implementation of OR as well as suggested regular revision of the agenda. Similar to the trend obtained in this study, recommendation for multi-disciplinary approach in the planning of MOR agenda as well as the implementation of OR have earlier been noted in other studies [5, 10, 15, 16]. The study suggested the need to create awareness and encourage collaborations among researchers and institutions as well as building capacity of young or upcoming researchers in the areas of MOR. This is in line with the WHO priority for implementation’ science, whereby postgraduate training in implementation science is being sponsored in selected universities in sub-Saharan Africa. The WHO Special Programme for Research and Training in Tropical Disease helps low- and middle-income countries to strengthen their capacity to conduct research and use research evidence while setting policies and strategies [17].

While this study involved several categories of malaria stakeholders including malaria researchers, medical consultants, programme implementers, development partners who were well-known and experienced; we did not capture those who did not have link with institutions or NMEP. They may have provided further insights on MOR.

## Conclusion

Though not commonly undertaken, MOR was perceived to be very important in giving directions to aspect of malaria control to focus on, in order to achieve malaria elimination. Training institutions and in-country human resource capacity for MOR exist but presence of an existing national framework to guide MOR is uncertain. University-based researchers, followed by programme implementers in NGOs were the major stakeholders that were involved in collaborative MOR. The MOR is rarely used to inform change in policy or practice and several needs exist for MOR across thematic areas. There is an urgent need to revise the existing framework for MOR if available, set an all-inclusive MOR agenda using systematic approach, disseminate this widely and review periodically. While researchers should continue to conduct MOR studies that meet the national needs, the NMEP should strive to get the findings into policy and practices towards effective malaria control and eventual pre-elimination. In addition, NMEP should endeavor to publish the revised malaria OR agenda and prioritised list of research topics to further guide researchers to embark on relevant studies.

## Supporting information

S1 Appendix Key informant interview guide. (PDF)

S1 Dataset Malaria OR agenda setting data. (HPR7)
