# Supplementary material for: A qualitative exploration of malaria operational research situation in Nigeria
Source: PLoS One. 2017 Nov 15;12(11):e0188128. doi: 10.1371/journal.pone.0188128 (PMC5687705; doi:10.1371/journal.pone.0188128)
Supplement: S1 Appendix — (PDF) [file pone.0188128.s001.pdf]

AGENDA-SETTING FOR OPERATIONAL RESEARCH
(NATIONAL MALARIA ELIMINATION PROGRAMME)

Key informant interview guide for key malaria stakeholders and policy makers

Respondent Code		Qualifications	
Organisation			
Position in organisation *(Ask at the end of interview)*		Years in this position	
Interviewers		Age	
Date		Gender	
Informed consent given		Role in malaria control	

Interviewers introduce yourselves:

Explain project:
The Nigeria Field Epidemiology and Laboratory Training Programme and National Malaria Elimination Programme are carrying out a study on Setting Malaria Operational Research (OR) agenda in Nigeria. This is to provide a structured approach for setting malaria OR agenda while updating and prioritising list of questions that enhance innovation in malaria control.
To achieve this goal, we are conducting interviews with key malaria stakeholders and policy makers. We want to explore the gaps/challenges and research needs for malaria operational research in Nigeria.

For the purpose of this exercise:

Operational research is a form of research you conduct because you are looking for solutions to problems. Often when you look at a system or disease [e.g. malaria], you may find that things are not going the way you expect them to go (i.e. there are gaps and challenges), so you conduct a research to find out what the issues are and proffer solutions/interventions to that. The research can also ascertain the feasibility, effectiveness, accessibility and the coverage of the intervention(s) [Please note, this definition can be shared with interviewee ahead of the interview. DO NOT SHARE THE KII GUIDE BEFORE THE INTERVIEW]

Interviewer

Before we start, we want to let you know that we will be recording this interview and it will be transcribed to ensure that all of the information that we document is accurate. Results from interviews will not specifically identify individuals interviewed or their organizations. Rather, the results will be reported in aggregate. Do you have any questions? Is it ok to begin the interview?

Questions.

1. Is there an existing framework that guides malaria operational research in Nigeria?
2. Are there capacities to identify country-specific needs for malaria OR? (Probe)
3. Are there strategies for implementing malaria interventions? (Probe: would you consider any of these innovative)
4. To what extent do stakeholders use evidence based information or research findings from researchers across the country

   Prompt:

   i. Do you think that policy makers are using the outcome of OR for policy reform?
   ii. What are the reasons for your response?
5. Could you share with us your experience working with malaria researchers in Nigeria if any? Have you participated or collaborated with a group?
6. Do you have any research collaborator with focus on malaria operational research?

   Prompts:

   i. If yes, what intervention has been or is being implemented?
   ii. What is the implementation model?
7. What are the operational research gaps identified from these research projects in Nigeria?
8. What are the challenges in malaria operational research identified from these research projects in Nigeria?
9. What do you think are the malaria operational research needs in Nigeria?
10. What informed the choice of malaria OR carried out?

    Prompt:

    i. Are you aware of NMEP operational research list?
    ii. if yes, does your organisation consider this in deciding the OR to conduct?
11. How many malaria OR has your organisation carried out (or in collaboration) since year 2010? How many in the last two years?

    Prompt:

    i. Does your organisation have budget for malaria OR?

ii. If yes, what percentage of your budget is for malaria OR?

12. How did you disseminate the findings of your OR research?

13. Do you think research agenda setting is important for malaria operational research

    Prompt:

    i. In setting malaria operational research agenda, what do you think should be the content? who should be involved?

    ii. What are the reasons for your response?

    i. What are your suggestions on the process to setting malaria operational research agenda?

14. What would you like to see in a National malaria operational research agenda?

Do you have any other comment or information you would like to share?

Thank you very much for your time and for sharing your experiences with us.

Have a good day.
